# Maternal and newborn health services utilization in Jimma Zone, Southwest Ethiopia: a community based cross-sectional study

**DOI:** 10.1186/s12884-019-2335-2

**Published:** 2019-05-22

**Authors:** Lelisa Sena Dadi, Melkamu Berhane, Yusuf Ahmed, Esayas Kebede Gudina, Tasew Berhanu, Kyung Hwan Kim, Masrie Getnet, Muluemabet Abera

**Affiliations:** 1 Department of Epidemiology, Faculty of Public Health, Jimma University, Jimma, Ethiopia; 2 Department of Pediatrics and Child Health, Jimma University Medical Center, Jimma, Ethiopia; 3 Department of Obstetrics and Gynecology, Jimma University Medical Center, Jimma, Ethiopia; 4 Department of Internal Medicine, Jimma University Medical Center, Jimma, Ethiopia; 5 Jimma Zone Health Office, Jimma, Ethiopia; 6 KOICA Ethiopia Center, Jimma, Ethiopia; 7 Department of Population and Family Health, Faculty of Public Health, Jimma University, Jimma, Ethiopia

**Keywords:** Antenatal care, Delivery services, Postnatal care, Jimma/Ethiopia

## Abstract

**Background:**

Majority of causes of maternal and newborn mortalities are preventable. However, poor access to and low utilization of health services remain major barriers to optimum health of the mothers and newborns. The objectives of this study were to assess maternal and newborn health services utilization and factors affecting mothers’ health service utilization.

**Methods:**

A community based cross-sectional survey was carried out on randomly selected mothers who gave birth within a year preceding the survey. The survey was supplemented with key informant interviews of experts/health professionals. Multivariable logistic model was used to identify factors associated with service utilization. Adjusted odds ratios (AORs) were used to assess the strength of the associations at *p*-value ≤0.05. The qualitative data were summarized thematically.

**Results:**

A total of 789 (99.1% response rate) mothers participated in the study. The proportion of the mothers who got at least one antennal care (ANC) visit, institutional delivery and postnatal care (PNC) were 93.3, 77.4 and 92.0%, respectively. Three-forth (74.2%) of the mothers started ANC lately and only 47.5% of them completed ANC_4_+ visits. Medium (4–6) family size (AOR: 2.3; 95% CI: 1.1, 4.9), decision on ANC visits with husband (AOR: 30.9; 95% CI: 8.3, 115.4) or husband only (AOR: 15.3; 95%CI: 3.8, 62.3) and listening to radio (AOR: 2.5; 95%CI: 1.1, 5.6) were associated with ANC attendance.

Mothers whose husbands read/write (AOR: 1.6; 95% CI: 1.1, 2.), attended formal education (AOR: 2.8; 95% CI: 1.1, 6.8), have positive attitudes (AOR: 10.2; 95% CI: 25.9), living in small (AOR: 3.0; 95% CI: 1.2, 7.6) and medium size family (AOR: 2.3; 95% CI: 1.2, 4.1) were more likely to give birth in-health facilities. The proportion of PNC checkups among mothers who delivered in health facilities and at home were 92.0 and 32.5%, respectively. The key informants mentioned that home delivery, delayed arrival of the mothers, unsafe delivery settings, shortage of skilled personnel and supplies were major obstacles to maternal health services utilization.

**Conclusions:**

Health information communication targeting husbands may improve maternal and newborn health services utilization. In service training of personnel and equipping health facilities with essential supplies can improve the provider side barriers.

**Electronic supplementary material:**

The online version of this article (10.1186/s12884-019-2335-2) contains supplementary material, which is available to authorized users.

## Background

Majority of the causes of maternal and newborn mortalities/morbidities are known to be preventable using basic interventions [1]. Curbing preventable maternal and newborn mortality is critical to achieving the Sustainable Development Goals (SDGs) [2]. In spite of the unreserved efforts made by the government and other partners, the maternal mortality ratio and infant mortality rate remain unacceptably high and among the unachieved Millennium Development Goal (MDGs) targets in Ethiopia. Studies show that the reduction in maternal mortality remains insignificant over the past three decades [3, 4]. It has been estimated that SDG 3.1 can be achieved if coverages of first antenatal care visit (ANC_1_), fourth antenatal care visit (ANC_4_), institutional delivery and skilled birth attendance reach 91, 78, 81, and 87%, respectively [5].

A health facility-based study conducted in central Ethiopia [6] revealed that in spite of high ANC_1_ (89.4%), institutional delivery (82.4%) and postnatal care (PNC) within six hours of delivery (74.6%), below half (46.6%) and only 14 (1.2%) of the study participants reported to have had three or more ANC and PNC visits, respectively. An earlier community-based study conducted in Northwest Ethiopia [7] also showed that only 12.1% of the mothers gave birth in health institutions during the 12 months preceding the survey.

Poor access to and low utilization of key interventions due to different socio-cultural and economic factors are still major obstacles to optimum maternal and newborn health [5, 8]. For instance, a study shows that among mothers who gave birth, only less than a third (32%) had reported to having attended the recommended ANC_4_+ visits [6, 9]. Other studies [2, 10] have also shown that women health care utilization is adversely affected by distance to health institutions, related costs, cultural barriers and customs, low status of women, husband disapproval, and low level of health awareness.

Studies conducted in Ethiopia [11], Ghana [12] and Nigeria [13] on Demographic and Health Surveys (DHSs) data also indicated the influence of contextual and individual factors, including maternal age, education, marital age, residence, wealth and access to media on mothers’ skilled delivery care utilization. Other studies [14–16] also noted that ANC_4+_, knowledge of pregnancy danger signs, place of delivery and delivery complications are significantly associated with PNC utilization of mothers. Still, other studies [12, 17, 18] show that maternal health services utilization is influenced by the educational status of the mothers/partners, household wealth, residence/distance to health facilities. On the other hand, service-related factors can greatly influence the health-seeking behavior of the mothers. Studies [19–22] show that perceived quality of services, inconsistent availability of medical supplies and unethical approaches or unavailability of trained care providers adversely affects the choice of place for maternal health services.

For instance, a study conducted at Assela Hospital, central Ethiopia, reported that mothers were dissatisfied due to poor cleanliness and lack of latrines while they were under intrapartum care [23]. Again, a study conducted in Addis Ababa shows that 78.0% of respondents experienced one or more categories of disrespect and abuse in health centers (75.3%) and hospitals (81.8%) during their previous visits [24]. Another hospital-based study conducted in Addis Ababa revealed that overall, only 19.0% of the mothers were satisfied with intrapartum care [25]. A similar study [26] reported that the level of satisfaction with intrapartum care was low. The main unsatisfying factors were poor pain management, lack of client privacy and client-provider communication.

Several studies [27–31] show that maternal health services utilization is influenced by multitude socioeconomic and demographic factors, which vary widely with the local context. According to a multilevel analysis of EDHS of 2011 [32], significant variations in the usage of antenatal and delivery care services were found to be attributed to an array of individual, household and context-related factors. This implies the need for monitoring level of maternal health services utilization in different settings in relation to locally influential factors. Therefore, this study has assessed the status of maternal health service utilization and factors affecting their health-services utilization.

## Methods

### Study setting and design

The study was conducted in selected districts of Jimma Zone, which is located 352 kms from the capital Addis Ababa in southwest of Ethiopia. According to projection of 2007 population census, the zone has an estimated population of 3,261,371 (49.9% women) in the year 2017. From the total females, 23.1% were women in the child bearing age (WCBA), which imply a total of 753,377 WCBA residing in the zone. The zone consists of 20 rural districts and one special town administration (Agaro Town), 46 small urban and 512 rural kebeles (smallest formal administrative units). This study was conducted from 06 to 18 September 2017 as part of maternal and neonatal intervention baseline assessment of the rural districts. This was supplemented by key informant interviews of heads/experts of the district health offices and health centers.

#### Study population/data source

The study population for the questionnaire-based surveys of this study was WCBA who gave birth within one year preceding the survey. The heads/experts of district health offices and heads/experts of the heath institutions that have been at the position at least for six months during the survey were included into the key informant interviews.

#### Sample size determination and sampling procedure

The data analyzed for this study are from a baseline study of maternal and newborn interventions, which has a control arm for the purpose of comparison after the intervention. Therefore, the sample size was determined using two-population proportion formula; considering 95% confidence interval, power of 80% to detect at least 10% difference between the two groups after the intervention. Based on previous community based study [7], the total sample size was estimated based on the study objective, using the variable which gives the largest sample size (#758) among the indicators of service utilization of WCBA. Assuming 5% non-response rate, the final sample size was estimated to be 796 respondents.

**Fig. 1:**
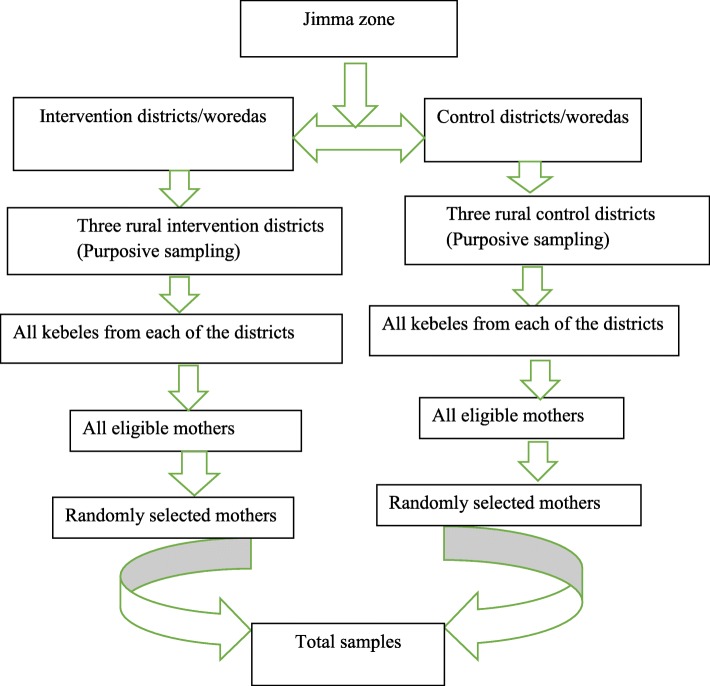
schematic presentation of sampling procedures

Initially six districts (30% of the 20 districts) were selected purposely considering absence of other maternal and newborn intervention projects and their representativeness in terms of agroecological climate and physical accessibility to health services. Simple random sampling technique was employed to recruit the interviewed WCBA for the community-based study. A fresh list of mothers who gave births during a year preceding the study was obtained from health extension workers and used as sampling frames. The sample size was allocated to each selected kebele proportionally to the size of WCBA who fulfilled the inclusion criteria. Finally, selection of the study subjects was made randomly and independently based on the prepared list within each kebele until the required sample size was achieved (Fig. 1).

Furthermore, in-depth interviews of 30% of heads/experts of public health institutions and all district health offices of the districts were done. Accordingly, key informant interviews of 11 experts from public health institutions and six heads/experts of district health offices of the study districts were carried out.

#### Data collection procedure

The WCBA data was collected by interviewer-administered questionnaire (Additional file 2) whereas interview guide (Additional file 1) was used for the in-depth interviews. Twelve (two per district) data collectors who hold bachelor degree in health science fields and who are trained for two days collected the survey data. Similarly, six master degree holders in health discipline supervised the quantitative data collection and conducted the qualitative study using guidelines prepared for these purposes. The data collection tools for the survey were pretested on 5% of the sample size outside the study setting before the commencement of the actual work. Sufficient training was given to data collectors and supervisors. In addition, the day-to-day activities of the data collection were closely supervised by the investigators. Data entry template was designed in EpiData version 3.1 to control error entries.

### Study variables

#### Dependent/outcome variables

- Maternal and newborn healthcare utilization: ANC, Institutional delivery and PNC services
  - **Antenatal care (ANC)** is the care provided by skilled health-care professionals to pregnant women in order to ensure the best health conditions for both the mother and the baby during pregnancy [33].
- **Institutional delivery** Institutional delivery means giving birth to a child in a medical institution under the overall supervision of trained and competent health personnel where there are more amenities available to handle the situation and save the life of the mother and child [34].
- **Postnatal care** (**PNC**) includes services provided to women and newborns immediately after delivery and up to six weeks thereafter, with the aim of ensuring optimum health for both the mothers and their infants [35]. In this study first PNC visit has been considered, which is consistent to EDHS 2016 [9].

#### Independent variables/predictors

- **Demographic characteristics**: age, parity, gravidity, marital status, ethnicity
- **Socioeconomic characteristics**: household economic status, availability and watching/listening of TV and radio; religion, educational status, occupational status, distance to health facilities
- **Others**: Respects given by health professionals, waiting time of clients, knowledge of pregnancy related danger signs

##### D**ata processing and analysis**

Data were checked, cleaned, and entered into EpiData version 3.1. Afterwards, data were exported and analyzed using SPSS® (IBM SPSS Statistics for Macintosh, Version 21.0. Armonk, NY). Both descriptive statistics and multivariable analytical model were used to summarize the data and identify association of independent variables with outcomes of the study, respectively. Based on a previous study definition [36], respondents who could correctly identify at least three key danger signs related to pregnancy, labor and delivery were considered to be knowledgeable of the key danger signs, or otherwise not. Adjusted odds ratios (AORs) with their corresponding 95% CIs were used to assess the strength of the associations at *p-value* ≤ 0.05 cut-off point for statistical significance. Additionally, qualitative data was transcribed and analyzed thematically.

## Results

A total of 789 (99.1% response rate) women in the childbearing age (WCBA) were included in the survey. The minimum and maximum ages of the study participants were 16 and 48 years, respectively. Close to half (47.9%) of them were in the age range 25 to 34 years, and the majority of them were Muslims (89.7%), Oromo ethnic group (87.2%), married (95.6%), housewives/ farmers (92.5%) and had not attended formal education (88.5%). Nearly half (49.7%) of them had a radio and most of those who had the radio (88.5%) reported that they listen to it regularly (Table 1).

**Table 1:** Socio-demographic characteristics of the respondents (WCBA), Jimma Zone, Oromia Regional State, Southwest Ethiopia September 2017 (*n* = 789)

Characteristics	Categories	Number (%)
Age during the survey	≤18	33 (4.2)
	19–24	255 (32.3)
	25–34	378 (47.9)
	35–49	123 (15.6)
Religion	Muslim	708 (89.7)
	Christian*	81 (10.3)
Ethnicity	Oromo	688 (87.2)
	Amhara	37 (4.7)
	Gurage	19 (2.4)
	Others	45 (5.7)
Marital Status	Married	754 (95.6)
	Not in marriage**	35 (4.4)
Educational status	Unable to read & write	393 (49.8)
	Only read and write	305 (38.7)
	Have formal education	91(11.5)
Do you have radio	Yes	392 (49.7)
	No	397 (50.3)
Radio listening (those who have radio)	Yes	347 (88.5)
	No	45 (11.5)

*Christian: Orthodox = 8.4%; Protestant = 1.5% & Catholic = 0.4%

**Not in marriage*: Separated = 2.3%; divorced = 1.4%; widowed = 0.5% & single = 0.2%*

Regarding the reproductive history of the study participants, almost half (49.7%) of them got married below the age of 18 years whereas close to another half (48.8%) of them got married between the age of 18 to 24 years. Again, a considerable proportion (45.4%) of the mothers got pregnant at or below the age of 18 years. More than half (58.2%) of them had two to five pregnancies and gave birth twice (52.9%) or more (5.7%); whereas 19.4% of the mothers reported having got more than five pregnancies. On the other hand, nearly one in ten (9.3%) of the mothers reported having experienced abortions and stillbirths (1.6%). Moreover, a quarter (25.3%) of them reported to have encountered health problems during their recent pregnancies (Table 2).

**Table 2:** Reproductive history of the respondents (WCBA), Jimma Zone, Oromia Regional State, Southwest Ethiopia, September 2017 (*n* = 789*)*

Variables	Categories	Number (%)
Age at first marriage	< 18 years	392 (49.7)
	≥ 18 years	397 (50.3)
Age at first pregnancy	≤18 years	358 (45.4)
	≥ 19 years	431 (54.6)
Number of life time pregnancies	1	177 (22.4)
	2–5	459 (58.2)
	> 5	153 (19.4)
Number of deliveries in the last 5 years	1	327 (41.4)
	2	417 (52.9)
	> 2	45 (5.7)
Abortion experienced	Yes	73 (9.3)
	No	716 (90.7)
Number of abortions ever occurred	1 time	60 (7.6)
	≥ 2 times	13 (1.6)
Number of still births ever occurred	1	9 (1.1)
	≥2	4 (0.5)
Health related problems encountered during last pregnancy	No	589 (74.7)
	Yes	200 (25.3)

Most (93.3%) of the study participants reported having attended antenatal care (ANC) during their last pregnancy, mainly (71.0%) starting at the second trimester. However, only 47.5% of them had attended the recommended ANC_4+_ visits per pregnancy. Majority (79.7%) of them made the ANC visits at health centers followed by health posts (15.0%); hence, a large proportion (83.4%) of the mothers reported to have been attended by skilled health professionals. A large proportion (72.9%) of the respondents reported to have received health information during the ANC visits though only half (50.8%) of them remembered to have been informed about danger signs of pregnancy. Majority (86.7%) of them reported to have been informed where to deliver; hence, more than three quarters (77.4%) of them gave birth at health institutions, which signify the importance of health information for pregnant mothers to strengthen maternal and newborn health services (Table 3).

**Table 3:** Maternal health service utilization of the respondents (WCBA), Jimma Zone, Oromia Regional State, Southwest Ethiopia, September 2017 (*n* = 789)

Variables	Categories	Number (%)
ANC visit	Yes	736 (93.3)
	No	53 (6.7)
Number of ANC visit	1	9 (1.1)
	2	74 (9.4)
	3	278 (35.2)
	≥ 4	375 (47.5)
Time of first visit	First trimester	149 (18.9)
	Second trimester	560 (71.0)
	Third trimester	25 (3.2)
	I don’t remember	2 (0.3)
Place of ANC visit	Health Centre	629 (79.7)
	Health post	118 (15.0)
	Hospital	33 (4.2)
	Private Clinic	6 (0.8)
ANC attendants	Health professional	618 (84.0)
	Health extension worker	118 (16.0)
Information received during ANC	Yes	684 (86.7)
	No	52 (6.6)
Given HE during ANC visits	Yes	575 (72.9)
	No	145 (18.4)
	I don’t remember	16 (2.0)
Informed about danger signs of pregnancy	Yes	401 (50.8)
	No	166 (21.0)
	I don’t remember	8 (1.0)
Informed where to deliver	Yes	684 (86.7)
	No	52 (6.6)
Place of delivery	Home	178 (22.6)
	Health Facility	611 (77.4)

Regarding postnatal care services, 92.0% of the mothers reported getting checkup while they were in the health institutions, half (50.3%) of them within a day of their delivery, a third (33.8%) of them at least six hours later after the delivery, yet the first checkups took place within six hours of delivery for three-fourth (76.4%) of them. The present study shows that about a third (32.5%) of those who gave birth at home had got checkups, but the rest did not get any professional support. The most noted postnatal services mentioned were advice on breastfeeding (56.1%) and family planning (40.1%), physical examination (34.6%) and provision of family planning services (22.3%).

Pertaining to the mothers’ knowledge of danger signs, the respondents knew some danger signs related to pregnancy (60.8%), labor & delivery (59.3%), and danger signs that can be manifested within first week after delivery (51.3%). The major cited danger signs related to pregnancy were vaginal bleeding (30.0%), severe headache (29.3%) and persistent vomiting (28.0). Similarly, danger signs associated with labor & delivery were mentioned to be vaginal bleeding (35.0%), prolonged labor (32.6%) and retained placenta (20.9%). Again, the main danger signs that can occur within one week after delivery were mentioned to be severe vaginal bleeding (38.5%) and high-grade fever (21.7%).

A total of 116 (14.7%) of the respondents reported that they have encountered health problems related to childbirth during their last deliveries. High-grade fever (32.8%), excessive vaginal bleeding (31.9%), and pain during urination (12.1%) were the common problems listed by the respondents. Nearly three-fourth (73.7%) of them sought treatment in the nearby health facility for their health problems. Regarding the attitude of husbands towards institutional delivery, 94.6% of the study participants said that their husbands were supportive and have a favorable attitude towards institutional delivery (Table 4)**.**

**Table 4:** Mothers’ knowledge of danger signs related to pregnancy and problems encountered after delivery, Jimma Zone, Oromia Regional State, Southwest Ethiopia, September 2017 (*n* = 789)

Variables	Categories	Number (%)
Knew danger signs related to pregnancy (51.1%)	Vaginal bleeding	291 (36.9)
	Severe headache	190 (24.1)
	Blurred vision	90 (11.4)
	Persistent vomiting	72 (9.1)
	Swollen hands/face	67 (8.5)
	Hypertension	41(5.2)
	Foul smelling vaginal discharge	29 (3.7)
	Other	30 (7.5)
	Knew ≥3 of the danger signs	111(14.1)
Knew danger signs related to labor and delivery (59.3%)	Vaginal bleeding	276 (35.0)
	Prolonged labor (> 12 h)	257 (32.6)
	Retained placenta	165 (20.9)
	Hypertension	55 (7.0)
	Others	52 (6.6)
	Knew ≥3 of the danger signs	59 (7.5)
Knew danger signs that occur within the first week after birth, # 405 (51.3%)	Severe vaginal bleeding	304 (38.5)
	High fever	171 (21.7)
	Fit	60 (7.6)
	Hypertension	45 (5.7)
	Swollen hands/face	20 (2.5)
	Offensive vaginal discharge	51 (6.5)
	Other	60 (7.6)
	Knew ≥3 of the danger signs	71 (9.0)
Health problem encountered after delivery	yes	116 (14.7)
	no	673 (85.3)
Sever vaginal bleeding	yes	37 (31.9)
	no	79 (68.1)
High grade fever	yes	38 (32.8)
	no	78 (67.2)
Painful urination	yes	14 (12.1)
	no	102 (87.9)
Offensive vaginal discharge	yes	4 (3.4)
	no	112 (96.6)
Do not remember		7 (6.7)
Support sought for the health problems encountered	Yes, at health facility	84 (73.7)
	Yes, at home	18 (15.8)
	Not at all	12 (10.5)
Husband’s attitudes towards institutional delivery	Supportive	746 (94.6)
	Not supportive	22 (2.8)
	Don’t know	21 (2.7)

Regarding delivery services, 599 (77.9%) study participants reported having received delivery services at the health facilities whereas the remaining 22.1% of the mothers delivered at home. The reported reasons for home delivery were perceived short labor duration (74.2%), long distance to health facilities (12.9%) and absence of delivery service (9.5%) in the nearby health facilities (health posts). Among those mothers who delivered at the health facilities, 58 (9.7%) reported to have paid for the services and out of whom the payment was costly for seven (12.1%) of them. Concerning distance to the health institutions where they received health care, majority (93.5%) of the interviewed mothers reported walking less than one hour to reach the nearest health facility. Close to a quarter (24.8%) of the respondents reported that they waited for long time to receive the services.

On the other hand, majority of the mothers responded that their privacies were maintained during intrapartum procedures (87.1%) and the health professionals were respectful to them (88.1%). The overall satisfaction level of the mothers with different components of the maternal health services was categorized to be very good (46.2%), good (37.2%), fair (10.3%) and poor (5.6%) or very poor (0.8%) (Table 5).

**Table 5:** Health facility related factors among women who delivered their last child in health institution, Jimma Zone, Oromia Regional State, Southwest Ethiopia, September 2017

Variables	Categories	N (%)
Received delivery service	yes	611 (77.4)
	No	178 (22.6)
Payment for the delivery services	Yes	58 (9.7%)
	No	540 (90.3%
Payment for the services	Too expensive	7 (12.1)
	Fair	39 (67.2)
	Cheap	8 (13.8)
	I don’t remember	4 (6.9)
Time to reach health facility	< 1 h	719 (93.5)
	1–2 h	50 (6.5)
Waiting time to get delivery service	Long	146 (24.8)
	Short	443 (75.2)
Health workers have confidence	Yes	542 (92.5)
	No	44 (7.5)
Privacy during the procedures	yes	513 (87.1)
	no	40 (6.8)
	I don’t know	36 (6.1)
Health workers were respectful	yes	519 (88.1)
	no	60 (10.2)
	I don’t know	10 (1.7)
Overall satisfaction level by the services	Very good	282 (46.2)
	Good	227 (37.2)
	Fair	63 (10.3)
	Poor	34 (5.6)
	Very Poor	5 (0.8)

### Factors affecting utilization of maternal healthcare services

The satisfaction levels (differences) did not show significant association with related independent variables. Husband educational status, age of mothers during the survey and at first marriage, distance to health facility and hearing information from TV did not show significant association with ANC utilization of the mothers.

On the other hand, age at first pregnancy, family size, decision-making pattern about ANC and getting information on the advantages of ANC showed at least marginally significant associations at multiple regression level analyses. There is significant association between age at first pregnancy and ANC attendance, where mothers who got their first pregnancy at the age of 19 years or above were (AOR: 2.6; 95% CI: 1.0, 6.5; *P* = 0.05) more likely to attend ANC compared to mothers who got pregnant before the age of 19 years.

Mothers who had 4 to 6 family members were more than two times (AOR: 2.3, 95% IC: 1.1, 4.9) more likely to make ANC follow-up compared to those mothers with more than six family members. Mothers who make decision with their husbands (AOR: 30.9; 955 CI: 8.3, 115.4) or whose husbands make decision (AOR: 15.3; 95%CI: 3.8, 62.3) on ANC attendance were more likely to make ANC follow-up compared to those mothers who make the decision themselves on ANC follow up. Mothers who get information on ANC benefits from radio were more than two times (AOR: 2.5; 95% CI: 1.1, 5.6) more likely to make ANC follow up compared to those mothers who did not listen to the radio (Table 6).

**Table 6:** Factors affecting ANC Utilization among women in the child bearing age group, Jimma Zone, Oromia Regional State, Southwest Ethiopia, 2017

Variables	Categories	ANC use		Odds Ratio (95% CI)		*P* Values (Adjusted)
		Yes	No	Crude	Adjusted	
Husband Education	Does not read/write	276	26	1	1	
	Read and write	327	18	1.7 (0.9, 3.2)	1.2 (0.6, 2.6)	0.56
	Formal educ.	104	3	3.3 (1.0, 11.0)	1.1 (0.3,4.3)	0.86
Age of mothers during the survey	≤18	29	4	1.1 (0.3, 3.5)	2.5 (0.3, 21)	0.40
	19–24	244	11	3.3 (1.5,7.4)	1.6 (0.5,4.8)	0.41
	25–34	356	22	2.4 (1.2,4.8)	1.8 (0.8, 4.0)	0.18
	35–49	107	16	1	1	
Age at first marriage	< 18	328	32	1	1	
	≥18	375	21	1.5 (0.9, 2.7)	0.6 (0.3, 1.7)	0.36
Age at first pregnancy	≤18	328	32	1	1	
	≥19	408	21	1.9 (1.1, 3.5)	2.6 (1.0, 6.5)	0.05
Distance to health facility	< 30 Minutes	302	12	2.2 (0.7, 7.1)	1.5 (0.4, 5.4)	0.58
	30–60 Minutes	375	30	1.1 (0.4, 3.2)	1.1 (0.3, 3.8)	0.88
	> 60 Minutes	46	4	1	1	
Family size	1–3	135	4	4.5 (1.6, 13.1)	3.3 (0.8, 14.1)	0.11
	4–6	384	20	2.6 (1.4, 4.6)	2.3 (1.1, 4.9)	0.04
	≥7	217	29	1	1	
Decision maker to seek ANC	Husband & wife	579	31	4.7 (1.9, 11.5)	30.9 (8.3, 115.4)	0.00
	Husband only	129	15	2.2 (0.8, 5.8)	15.3 (3.8, 62.3)	0.00
	Wife herself	28	7	1	1	
Information from radio	Yes	316	11	2.9 (1.5, 5.7)	2.5 (1.1, 5.6)	0.03
	No	420	42	1	1	
Information from TV	Yes	130	2	5.5 (1.3, 22.8)	_	0.99
	No	606	51	1	1	

Factors like maternal education, age of mothers (at first marriage, at first pregnancy and during the survey), distance to nearest health facilities, hearing information from radio about institutional delivery, decision-making pattern to seek institutional delivery, and owned land size did not show significant association with utilization of institutional delivery at multiple logistic regression levels.

On the other hand, husbands’ education, number of pregnancies, family size, hearing information from TV on the befits of institutional delivery and attitudes of husbands towards institutional delivery showed significant associations (at varying degree) with attendance of institutional delivery. Mothers whose husbands can read/write (AOR: 1.6; 95% CI: 1.1, 2.5) and who have formal education (AOR: 2.8; 95% CI: 1.1, 6.8) were more likely to give birth at health institutions. Mothers with family sizes 1 to 3 (AOR: 3.0; 95% CI: 1.2, 7.6) and 4 to 6 (AOR: 2.3; 95% CI: 1.2, 4.1) were more likely to give birth at health institutions compared to mothers whose family size is greater than six. Mothers who get information from TV on benefits were more than two times (AOR: 2.1; 95% CI: 1.0, 4.4; *P* = 0.04) more likely to make ANC follow-up compared to those mothers who did not get information from TV. Finally, mothers whose husbands have a positive attitude towards institutional delivery were ten times (AOR: 10.2; 95%CI: 4.0, 25.9) more likely to give birth at health institutions (Table 7).

**Table 7:** Factors affecting place of institutional delivery among mothers, Jimma Zone, Oromia Regional State, Southwest Ethiopia, September, 2017

Variables	Categories	Place of delivery		Odds Ratios (95% CI)		*P* Values (Adjusted)
		HF	Home	Crude	Adjusted	
Mother Education	Illiterate	276	117	1	1	
	Read &write	251	54	2.0 (1.4, 2.9)	1.2 (0.7, 1.9)	0.517
	Formal educ.	84	7	5.1 (2.3, 11.3)	2.3 (0.8, 6.8)	0.127
Husband Education	Illiterate	202	100	1	1	
	Read & write	281	64	2.2 (1.5, 3.1)	1.6 (1.1, 2.5)	0.020
	Formal educ.	98	9	5.4 (2.6, 11.1)	2.8 (1.1, 6.8)	0.025
Age of mothers during survey	≤18	31	2	6.7 (1.5, 29.3)	8.3 (1.4, 51)	0.024
	19–24	210	45	2.0 (1.2, 3.3)	1.4 (0.6, 2.9)	0.022
	25–34	284	94	1.3 (0.8, 2.0)	1.1 (0.7, 2.1)	0.429
	35–49	86	37	1	1	
Number of pregnancy (Gravidity)	1	154	23	2.7 (1.6, 4.5)	0.4 (0.1, 1.2)	0.090
	2–4	283	86	1.3 (0.9, 1.9)	0.4 (02, 0.7)	0.006
	≥ 5	174	69	1	1	
Age at first pregnancy	≤18	259	101	1	1	
	≥19	352	77	1.8 (1.3, 2.5)	1.8 (0.9, 3.1)	0.057
Distance to health institutions	< 30 Minutes	276	38	2.8 (1.4, 5.7)	2.2 (1.0, 4.8)	0.054
	30–60 Minutes	289	116	1.0 (0.5, 1.9)	1.0 (0.5, 2)	0.929
	> 60 Minutes	36	14	1	1	
Family size	1–3	124	15	4.1 (2.2, 7.4)	3.0 (1.2, 7.6)	0.024
	4–6	322	82	1.9 (1.3, 2.8)	2.3 (1.2,4.1)	0.008
	≥ 7	165	81	1	1	
Decision maker	Husband & wife	483	127	1.5 (0.7, 3.3)	1.3 (0.4, 4.7)	0.680
	Husband only	103	41	1.0 (0.4, 2.3)	1.0 (0.3, 3.4)	0.978
	Wife herself	25	10	1	1	
Information from radio	Yes	260	67	1.2 (0.9, 1.7)	0.8 (0.5, 1.2)	0.325
	No	351	111	1	1	
Information from TV	Yes	120	12	3.4 (1.8, 6.3)	2.1 (1.0, 4.4)	0.040
	No	491	166	1	1	
Age at first marriage	< 18	289	102	1	1	
	≥ 18	320	76	1.5 (1.1, 2.1)	0.9 (0.5, 1.6)	0.718
Land size owned by HH	≤ 1 ha	40	588	1	1	
	> 1 ha	13	148	0.5 (0.4, 0.8)	0.7 (0.4, 1.0)	0.063
Husband attitude towards HF delivery	Supportive	36	710	5.4 (2.9 10.1)	10.2 (4.0, 25.9)	0.000
	Not supportive	17	26	1	1	
Number of parities within five years	1	274	53	1.9 (1.3, 2.6)	1.4 (0.8,2.3)	0.236
	≥ 2	337	125	1	1	

Most (92.0) of the mothers who gave birth in a health facility, reported having had PNC checkups, mainly (78.9%) within six hours of delivery. However, only close to a third (32.5%) of those mothers who delivered at home reported having PNC checkups. According to the findings from key informant interviews, the main maternal and newborn health problems were unsafe delivery settings, which lead to hypothermia, birth asphyxia, neonatal sepsis, and febrile illness. Infectious diseases such as pneumonia, diarrhea, measles, and undernutrition were mentioned to be the major killers of infants. Poor health-seeking behavior, including home delivery and delayed arrival of the clients to health facilities, were mentioned to have exposed mothers to excessive bleeding and other complications, including fistula in some instances.

The key informants explained that the major challenges in implementation of maternal and newborn health services in their respective districts were found to be low number of trained personnel/training opportunity; lack of transportation, including poor infrastructure and long distance to reach health facilities; delayed health seeking, husband’s influences on some of the services such as low utilization of long-acting contraceptives; low performance of health developmental armies at community level; lack/interruption of power supply and lack of functional refrigerators. Shortage of drugs and supplies were also cited to be major limitations from the service providers’ side.

The key informants also discussed the prevailing common practices of maternal and newborn health services. They mentioned that maternal waiting areas have been arranged at respective health centers. The community uses traditional ‘stretcher’ called ‘Siree’ to carry mothers to accessible places where they get ambulances to reach health facilities. Local women contribute money (One Ethiopian Birr for one mother) & different raw items used to prepare food as part of the birth preparedness of pregnant mothers. HEWs perform most of the PNC activities such as counseling & vaccinations at their respective catchment areas. One mentioned harmful traditional practice was an early marriage where girls can be married at age as low as 14 years.

## Discussion

Health check-ups during pregnancy, institutional delivery, and postnatal care are considered to be the most effective health interventions for preventing maternal and newborn morbidity and mortality, particularly in places where the general health status of the women is poor [37–39]. This study tried to assess the status of maternal health care service utilization and factors influencing utilization of the services among mothers who gave birth in the last one year prior to the survey, in six districts of Jimma Zone Oromia Regional State, Southwest Ethiopia. The study also tried to see the knowledge and attitude of mothers on ANC, delivery and PNC services.

This study shows that 93.3% of women visited health facilities at least once during their recent pregnancy for antenatal care. However, only 47.5% of them had attended the recommended four visits. This result is higher than studies conducted in other parts of the country: in Eastern Ethiopia, only 10% of women attended four ANC visits [40], in Dejen and Aneded districts, Northwest Ethiopia, only 12% of the women reported to have attended the recommended four ANC visits [41] and the 2016 EDHS report for Oromia Region shows only 50.7% of the mothers attended at least one ANC visit [9]. The high coverage of ANC service in the area might have been attributed to the current massive health promotion activities on maternal and child health issues by the government and other developmental nongovernmental organizations, which might have also increased awareness and health seeking behavior of the respondents and accessibility of the services.

With regard to the timing of the first ANC visit, this study shows that more than two-third (70%) of women went to a health facility for ANC in the second trimester of their pregnancy whereas, less than one fifth (18.9%) of them visited health facility during the first trimester. The finding is consistent with previous studies done in Northwest Ethiopia [42] and in Eastern Ethiopia [40]. However, it is less than a study conducted in Nigeria [43], where 28% of the pregnant mothers visited health facilities during the first trimester. There were marginally significant associations between age of the mothers at first pregnancy, family size, decision-making pattern on ANC visits as well as getting information from radio and ANC attendance of the mothers. In contrast, studies done in Eastern [40, 44] and Northwest Ethiopia [41] revealed that ANC utilization of mothers is affected by maternal socio-demographic factors such as educational status, marital status, urban-rural residence, distance to health facilities, plan of pregnancy, history of abortion and illnesses, and knowledge of pregnancy complications.

According to the present findings, a significant proportion (95.6%) of the mothers reported that they were not vaccinated against tetanus during ANC visits. This contrasts other studies, which reported 77.6% [45] and 72.5% [46] two or more tetanus vaccination (TT_2_+) among the study participants. The possible reason behind this fact could be more mothers might have completed the vaccination prior to their recent ANC visits and the health professional might also have known this fact as the vaccination cards are usually kept at the health facilities.

1This study revealed that more than three-fourth (77.4%) of the respondents reported having delivered their last child in health facilities. The main predictors of institutional delivery were husband’s education and supportive attitude and small family size of the mothers. A similar finding was observed in a recent study done in southern Ethiopia, in which 78.3% of women delivered their last child in the health facility [47], where maternal age, education, religion, occupation, access to information, residence, knowledge of danger signs and ANC visits were mentioned to be predictors of institutional delivery. However, the finding was higher than studies done in different parts of the country and compared to the national EDHS 2016 report, where only 26% of the deliveries in Ethiopia and 18.8% in Oromia Region took place at health facilities. Other studies done in Dodota [19] and Goba [46, 48] districts, both southeastern Ethiopia, reported similar findings that only 18.2 and 47% of the mothers delivered their last child in health institutions, respectively.

The possible explanations for the higher service utilization might be due to high commitment of the government, which requires most kebeles to be home delivery free owed to the expansion of the health extension programs and training of health development army, which improves the utilization of health services by linking community and health facilities, particularly health centers. According to a recent assessment conducted by Ethiopian Public Health Institute (EPHI) and partner institutions [49], almost all governmental health institutions offer normal delivery services.

The present study shows that 92.0% of the study participants have some postnatal care services. This result is higher than the findings of studies conducted in South Ethiopia [50], West Ethiopia [51], Rwanda [52] and Kenya [53], where those who got postpartum services constituted 51.4, 20.2, 47.9, 12.8, and 46.2%, respectively. The major predictors of PNC were mentioned to be awareness of obstetric danger signs, mothers’ autonomy of decision-making, four or more ANC visits, institutional delivery, secondary level education, better incomes, and employment status of the mothers. A systematic review based study [54] on PNC services in developing countries reported an average of 36.0% PNC utilization, where most of the aforementioned factors were also found to be significantly associated with the PNC attendance.

The present study shows that overall satisfaction of the mothers with the maternity services was judged to be very good (46.2%) and good (37.2%), implying 83.4% of the mothers were satisfied with the services provided to them. This is in contrast to the finding of a study conducted in Addis Ababa [25], where only 19.0% of the mothers reported satisfaction with MNCH services. Such gap in satisfaction level might be due to perception difference as women in the towns are more educated and expected to judge the expected level of care and supports to be provided by health professionals. Studies conducted in Addis Ababa [26] and Accra [55] confirm this fact that higher level education, better income, and urban residence were the predictors of dissatisfaction; moreover, perceptions of clinical competency and emotional supports affect satisfaction status of mothers.

This study has some limitations. Obviously, social desirability bias might have affected responses of both the mothers and the key informants in favor of wanted behaviors. There is also the possibility of recall bias, thus the study participants might have not correctly addressed past events in this regard. As the study is cross-sectional, it may not reflect the whole picture of maternal health services utilization status and influencing factors, particularly seasonal dependent factors.

## Conclusions

Health care attendance of the mothers during pregnancy was fairly high; however, most of the mothers started ANC lately and had not completed the recommended number of ANC visits. Although, both institutional delivery and postnatal care services were high, there were still some mothers who delivered at home without the support of skilled attendants. Husband education, attitude of the husbands and family size of the mothers were factors significantly affecting institutional delivery. Poor health-seeking behavior, including home delivery and delayed arrival of the mothers, were mentioned to have exposed mothers to excessive bleeding and other complications. Shortage of medical supplies was also cited as rate limiting factor.

Therefore, maintaining/improving the witnessed high-level of newborn and maternal health services by increasing awareness and health seeking behavior of the community using well-designed and locally contextualized IEC/BCC strategy is important. Improving capacity of the health facilities, including the provision of standard in-service training to the health personnel, and fulfilling essential equipment and supplies to ensure optimum newborn and maternal health services can improve the maternal health service utilization. Reaching mothers who are still inaccessible due to distance and other barriers to benefit them from newborn and maternal health services by facilitating transportation optimizing outreach health services and increasing scope of health information communication need due attention.

## Additional files

Additional file 1: Key informant interview guide: (PDF 411 kb)

Additional file 2: **Survey questionnaire:** WCBA_HHQ1_Base_End. The questionnaire has been developed and customized mainly from the Ethiopian Health and Demographic Survey (EDHS) report, maternal health section: https://dhsprogram.com/publications/publication-fr328-dhs-final-reports.cfm We have uploaded the PDF of the questionnaire as an additional file. (PDF 165 kb)

## Abbreviations

ANC: Antenatal care
AOR: Adjusted odds ratio
BCC: Behavioural change communication
CBNC: Community based neonatal care
EDHS: Ethiopian demographic and health survey
EPHI: Ethiopian public health institute
FMOH: Federal ministry of health
HEWs: Health extension workers
ICCM: Integrated community case management
IEC: Information, education and communication
IMNCI: Integrated management of neonatal and child illnesses
KOICA: Korea international cooperation agency
MDGs: Millennium development goals
MNCH: Maternal, neonatal and child health
PNC: Postnatal care
SDGS: Sustainable development goals
TT: Tetanus toxoid
WCBA: Women in the childbearing age
WHO: World health organization
