# Supplementary material for: Maternal and newborn health services utilization in Jimma Zone, Southwest Ethiopia: a community based cross-sectional study
Source: BMC Pregnancy Childbirth. 2019 May 22;19:178. doi: 10.1186/s12884-019-2335-2 (PMC6530050; doi:10.1186/s12884-019-2335-2)
Supplement: Supplementary file 1 — Key informant interview guide: (PDF 411 kb) [file 12884_2019_2335_MOESM1_ESM.pdf]

Jimma University

Implementation study to improving maternal and child health in Jimma zone Ethiopia.

In-depth Interview guide for heads/experts of public health care institutions

1. What are major health problems of mothers and children in the community/catchment area?
2. What are the common practices for care of pregnant woman in this community? **Probe**: ANC, TT, Food taboos, Work load, Decision making role of women, men and Mother in laws, preference of place of delivery.
3. What are the common preparations before the delivery? **Probe**: Preparation for money, person to assist in delivery and cord cutting, blood donor, place to delivery, food for delivered mother, cloths for newborns.
4. What are the common practices during delivery? **Probe**: Who actually assists in delivery? Role of TBAs, role of husband, role of mother in law, if delivered at home: where?
5. What are the common practices for newborn immediately after birth? **Probe:** Where to put the newborn? How to cut the cord? What to apply on the cord? How to clean the newborn? When to give bath? When to initiate breastfeeding? Do they feed colostrums or throw some colostrum? Immunization practice during newborn period?
6. What are the common practices for postnatal mother immediately after birth? **Probe:** Contact with newborn? Food and other care? Hygiene? Postnatal check up?
7. What are the major health problems among newborns? How do the mothers identify danger signs that require medical treatment? Where do they seek care first?
8. What do they do if the baby does not cry or does not breathe immediately after birth?
9. What are the services delivered by health facilities for mothers during pregnancy, delivery and postnatal period? How frequently do people use those services? Why or why not?
10. What community behaviors and practices should be discontinued or discouraged to improve health of mothers and newborn in this community?
11. What community behaviors and practices should be promoted or continued to improve health of mothers and newborns in this community?
12. What are the major challenges in providing maternal and child health services in your district?
13. Is there any ongoing health project in your catchment? If yes on what issues?
14. How do you rate the quality of data produced by the Health Management Information system of your organization? Can you explain more? Why?
15. Can you please mention and explain some of the challenges with your Health Management Information System?
