# Supplementary material for: Maternal and newborn health services utilization in Jimma Zone, Southwest Ethiopia: a community based cross-sectional study
Source: BMC Pregnancy Childbirth. 2019 May 22;19:178. doi: 10.1186/s12884-019-2335-2 (PMC6530050; doi:10.1186/s12884-019-2335-2)
Supplement: Supplementary file 2 — Survey questionnaire: WCBA_HHQ1_Base_End. The questionnaire has been developed and customized mainly from the Ethiopian Health and Demographic Survey (EDHS) report, maternal health section: https://dhsprogram.com/publications/publication-fr328-dhs-final-reports.cfm We have uploaded the PDF of the questionnaire as an additional file. (PDF 165 kb) [file 12884_2019_2335_MOESM2_ESM.pdf]

Data collections tools

Annex I:-Questionnaire for Women in the Child Bearing Age

Participant information sheet and consent form

My name is ________________________. I am here to conduct an assessment for improving maternal and child health in Jimma zone Ethiopia. You are selected to participate in this study randomly. The information you provide will contribute a lot for improving maternal and child health in your locality, further helps for a planner and for program designer who are working in the government as well as for Nongovernmental organization to designing an appropriate and cultural acceptable maternal and child health intervention to improve service utilization. The interview takes an average of an hour to complete. Whatever information you provide will be kept strictly confidential and will not be shown to other persons. Your name will not be written on this form, and will never be used in connection with any of the information you tell to us. Participation in this survey is voluntary and you can choose not to answer any individual question or all of the questions. However, we hope that you will participate fully in this study since your views are important.

Are you willing to participate in the study? Yes ____ No ____ Ok thanks

Yes, may I begin the interview now? YES –Continue

General Information

01. Respondent Identification number:__________02. Woreda: ____________________________

03. Kebele: ______________________________04. (Got/Zone)____________________________

05. House number ______________

06. Date of interview: ____________time interview started _____________ ended at _____________

07. Respondent available on: 1st visit_______ 2nd visit__________ 3rd visit___________ (Mark: / or X).

08. Status of interview (Circle): 1. Completed. 2. Partially completed (refused in the middle).

3. Refused: 4. Candidate was absent in 3 visits:

09. Interviewer's name:_________________________________ Signature: ______________

10. Supervisor who checked questionnaire for completeness and accuracy:

Name: _______________________________ Signature:______________________________________

Date: ____

Part I Socio-Demographic Characteristics of Respondents

Ser. No	Questions	Responses	Skip to	Coding
101	Age in complete year	_____________ Years		
102	What is your ethnic group?	1.Oromo 2.Amhara<br>3. Gurage 4. Tigrie<br>5.Other (specify)______________________		
10<br>3	What is your religion?	1.Orthodox 2.Muslim<br>3.Catholic 4.Protestant<br>5.Other (specify) __________________		
10<br>4	What is the highest education level you have attained?	1.Illiterate 2.Only read and write (no formal education)<br>3. Highest grade completed _________________		
10<br>5	What is your occupation?	1. Housewife 2. Farmer<br>3. Governmental employee 4. Merchant<br>5. Housemaid<br>6.Other (specify) __________________________		
106	What is your marital status?	1.Not married<br>2. Married<br>3.Divorced<br>4.Separated<br>5.Widowed<br>6.Other (specify) _______	Q 110	
10<br>7	Your husband's age	__________________		
108	Your husband's occupation	1. Governmental employee 2. Merchant<br>3. Farmer 4. Other (specify)		
10<br>9	Your husband's educational status	1.Illiterate 2.Only read and write (no formal education)<br>3.highest grade completed __________________		
11<br>0	Family size	__________________		
111	Do you have radio in your house?	1. Yes<br>2. No		
112	If yes to Q 111, do you listen to the radio?	1. Yes<br>2. No		
113	Do you have mobile phone?	1. Yes<br>2. No		
114	If "yes" for question number Qs113,<br>Do you have a direct communication with HEW?	1. Yes<br>2. No		
115	If "yes" for question number Qs114, tell us why you call them and by what frequency you contact them	1. Reason to calling ________<br><br>2.. Frequency of calling for HEW per a day________		
116	The number of living sibling (Kinship relationships)?	________________		
117	Do you have your own farm land and	1. Yes		

	grazing land?	2. No		
118	If Yes how much land size (in hectare)?	________________		
119	What are the means of transportation in your locality?	1. On foot<br>2. Bajai<br>3. Bicycle<br>4. Animal drawn truck<br>5. Motor cycle<br>6. Car/truck		
120	Do you have electricity access	1. Yes<br>2. No		
121	Household assets			
	• radio	1.<br>es number (_____)<br>2. No		
	• television	1. Yes -------number(_____)<br>2. No		
	• none mobile telephone	1. Yes ------number(______)<br>2. No		
	• refrigerator	1. Yes ------number(______)<br>2. No		

Part II: Reproductive History of Respondent

er. No	Questions	Responses	Skip to	Coding
**Now I would like to ask you some questions about your reproductive life.**				
201	What was your age when you were first married?	1. _________ Yrs<br>2. I don't remember		
202	What was your age at your first pregnancy?	1. _________ Yrs<br>2. I don't remember		
203	Number of pregnancies	______________		
204	Number of children born alive			
205	Number of still birth			
206	Number of new born who Died within seven days			
207	Number of new born who Died b/n 7days and 28 days.			
208	Live birth survived to28 days >1yr.			
209	Number of abortion			
210	How many times have you given birth to a child in the past five years?	___________________		
211	How many of them were delivered at home?	1. Live birth number______<br>2. Still birth number ___		
212	How many of them were delivered in health institution?	1. Live birth number______<br>2. Still birth number __________		
213	When was your last child born?	____________/________<br>Month year		

214	Have you ever heard health information about skilled care through radio?	1. YES<br>2. NO		
215	How often do you hear health information about skilled care on radio?	1. Once 2. Twice 3.three<br>4. four 5.five and more		
216	Have you ever heard or watch health information about skilled care on television?	1. YES 2. NO		
217	How often do you hear or watch health information on television?	1. Once 2. Twice 3.three<br>4. four 5.five and more		

Part III: Maternal Health Service Utilization

Ser No	Questions	Response	Skip to	Coding
301	Did you face any health problem in the last pregnancy	1. Yes<br>2. No		
302	If yes to Q 301, what were they?<br>(*Do not read the choices*)	1. Excessive Vaginal bleeding<br>2. Severe head ache<br>3. Face/ hand swelling<br>4. Persistent vomiting<br>5. Premature labor<br>6. Hypertension<br>7. Convulsion<br>8. Other (specify)_______________		
303	Did you visit a health facility during the last pregnancy?	1. Yes<br>2. No		
304	If yes to Q303, reason for visit	1. Pregnancy related health problem<br>2. Health problems not related to pregnancy<br>3. For antenatal care<br>Other (specify) --------------------------		
305	Did you receive any antenatal care during the last pregnancy?	1. Yes<br>2. No →	320	
306	If yes to Q 305, at what gestational age did you start pregnancy check up?	1. One to three months<br>2. Four to six months<br>3. Seven to nine months<br>4. I don't remember		
307	How many times did you go for pregnancy check up?	______________________________		
308	To which institution did you go for Antenatal care service?	1. Hospital<br>2. Health center<br>3. Health post<br>4. Private clinic<br>Other (specify) ________________		
309	Why did you prefer this institution?<br>(don't read the options)	1. Close to my house<br>2. Competent health worker<br>3. Fair price<br>4. To avoid long waiting time<br>5. Privacy issue<br>6. Other (specify) _________		
310	Who provided you antenatal care in	1. Health Extension Worker		

	the last pregnancy?	2. Health professional<br>3. Other (specify)___________		
311	Was health education given during each visit?	1. Yes, always<br>2. Yes, sometimes<br>3. Not at all<br>4. Don't remember		
312	If yes to Q 311 were you informed about danger signs related to pregnancy	1. Yes<br>2. No<br>3. Don't remember		
313	If Yes to Q 312, Which danger signs were you informed about?<br>(Don't read the choices)	1. Vaginal bleeding<br>2. Severe head ache<br>3. Face/ hand swelling<br>4. Persistent vomiting<br>5. Hypertension<br>6. Foul smelling vaginal discharge<br>7. Blurred vision<br>8. Other (specify)_________________		
314	Where you informed about where to deliver your baby?	1. Yes<br>2. No		
315	If yes to Q 314, where were you recommended to deliver?	1. Home<br>2. Health facility<br>3. Other (specify) _________________		
316	Were you informed about who should attend you during delivery?	1. Yes<br>2. No		
317	If yes to Q 316, who was recommended to attend your delivery?	1. Trained traditional birth attendant<br>2. Relative<br>3. Health professional<br>4. Other (specify)___________		
318	Were you given an injection in the arm to prevent you getting tetanus (Use Local Term For Tetanus)?	1. Yes<br>2. No ——————→	321	
319	If yes to Q 318, how many times?	1. Once<br>2. Twice<br>3. Three and above		
320	Do you have the card<br>(From the card register date of injection)	1. TT_1 ____/____/________<br>Date month year<br>2. TT_2 ____/____/________<br>Date month year<br>3. TT_3 ____/____/________<br>Date month year<br>4. TT_4 ____/____/________<br>Date month year<br>5. TT_5 ____/____/________<br>Date month year		

321	In general, Did you get the following service during your ANC visit?	**1. Yes**	**2. No**		
	• Weighed check				
	• Check blood pleasure				

	• Received abdominal examination				
	• Listened to baby's heartbeat				
	• Asked about medical history				
	• Provided a urine sample				
	• Advised on what to do for potential problem				
	• Asked to take, or took, a Syphilis test				
322	If you did not attend antenatal care, can you tell me the reasons? (Multiple answer is possible) **(Do not read the choice)**	1. No or little Knowledge about ANC<br>2. No health problem encountered<br>3. Health institution is too far from my home<br>4. Expense to ANC is unaffordable.<br>5. long waiting time<br>6. Poor handling by health care providers<br>7. Lack of transportation<br>8. Lack of time to go to health institution<br>9. Facilities not open regularly<br>10. No female health care provider<br>11. Husband un willingness<br>12. Lack of accompanies<br>13. presence on TBA<br>14. Other (specify)---------------------			
323	Where did you deliver your last child?	1. Home<br>2. Hospital<br>3. Health center<br>4. Health post<br>Other specify _________			
324	Who assisted you in the last delivery?	1. Health Extension Worker<br>2. Health professional<br>3. Neighbor,<br>4. Relative<br>5. Other (specify)___________			
325	For how long were you in labor during the last delivery?	1. Less than 12 Hrs<br>2. 12- 24Hrs<br>3. 25- 36 Hrs<br>4. 37 – 48 Hrs<br>5. More than 48 Hrs			
326	If your last delivery is in health institution, when did you go to the health institution?	1. At the beginning of labor<br>2. 6-12 hours after the beginning of labor<br>3. 13-18 hours after the beginning of labor<br>4. 19-24 hours after the beginning of labor<br>5. Other specify __________			
327	If your last delivery is in health institution, what was the mode of delivery?	1. Spontaneous vaginal delivery<br>2. Instrumental delivery<br>3. Caesarian section<br>4. Other (specify) __________			
328	What was the condition of the baby at birth in the last delivery?	1. Born alive<br>2. Still birth (born Died)<br>3. Born alive but died immediately			

329	If you had delivered your last pregnancy at home, why did you prefer to deliver at home?<br><br>(Don't read the choice)	1. The labor was short<br>2. No nearby health facility<br>3. The service is not available in the nearby health facility<br>4. Lack of money for service<br>5. Lack of money for transport<br>6. Poor handling by health professionals<br>7. Prefer to deliver in the presence of relatives<br>8. Fear of manipulation (like episiotomy)<br>9. Lack of privacy in the health institutions<br>10. I didn't know the importance of health facility delivery<br>11. Opinions of (husband, neighbors, other community members)<br>11. Other (specify) ____________		
330	Have you encountered any health problems during labor in the last delivery?	1. Yes<br>2. No<br>3. I don't remember		
331	If yes to Q 330, what were the problems?	1. Massive vaginal bleeding<br>2. Prolonged labor more than 12 hrs<br>3. Unconsciousness<br>4. Other (specify) ________		
332	Have you encountered any health problems during delivery when you deliver your last child?	1. Yes<br>2. No<br>3. I don't remember		
333	If yes to Q 332, what were the problems?	1. Massive vaginal bleeding<br>2. Retained placenta (more than 30 min)<br>3. Inability to control urine and faces<br>4. Birth canal laceration<br>5. Unconsciousness<br>6. Other (specify) ________		
334	If you delivered in the health facility How long after delivery did you stay there?	1. ________ hours<br>2. ______days<br>3. ______weeks		
335	Did anyone check on your health while you were still in the facility?	1. Yes<br>2. No		
336	If yes to question 335 How long after delivery did the first check take place	1. ________ hours<br>2. ______days<br>3. ______weeks		
337	For those who delivered at home<br><br>Did anyone check on your health after you gave birth	1. Yes<br>2. No		
338	If yes to question ___ How long after delivery did the first check take place	1. ________ hours<br>2. ______days<br>3. ______weeks		
339	Where did the first check take place	1. Home<br>2. Health facility<br>3. Other specify _________		

340	Who checked on your health at that time	1. Doctor<br>2. Nurse<br>3. Midwife<br>4. Health officer<br>5. Health extension worker<br>6. Others specify _______________		
341	If “Yes” What postnatal services did you receive when you went back to hospital after delivery?	1. Physical examination of mother<br>2. Counseling for family planning<br>3.Family planning services<br>4. Breast feeding education<br>5. Other (specify)___________________<br>_________________________________		
342	Did you face any health problem after your last delivery?	1. Yes<br>2. No		
343	If your answer is “yes” for question Q342<br><br>What were the problem are(don’t read)	1. Severe vaginal bleeding<br>2. High grade fever<br>3. Painful urination<br>4. Offensive vaginal discharge<br>5. Hot, swollen, painful breasts<br>6. Other (specify) ------------------<br>7. Don’t remember		
344	Where do look for help after you experience the above problem?	1. No help seeking<br>2. Help at home<br>3. Help gain at health institution.		
345	What was your husband’s attitude towards institutional delivery?	1. supportive<br>2. not supportive<br>3. Don’t know		
346	Who is the decision maker in your household to seek care from modern health institution?	1. Both of us<br>2. My husband<br>3. My self<br>4. Other (specify) _____________		

Part IV: Respondent Knowledge & Attitude to MCH Utilization

S. No	Question	Responses	Skip to	Coding
	**A. Knowledge questions**			
401a	Do you know any health problems related to pregnancy?	1. Yes<br>2. No →	404a	
402a	If yes to Q 401, can you mention some of the problems?<br>(Don't read the lists)	1. Vaginal bleeding<br>2. Severe headache<br>3. Hypertension<br>4. Convulsion<br>5. Persistent vomiting<br>6. Swollen hands/face<br>7. Other (specify)		
403a	In your opinion, could a woman die due to the mentioned problems?	1. Yes<br>2. No<br>3. Don't Know		
404a	Do you know any problems related to labor and delivery?	1. Yes<br>2. Don't know →	407a	
405a	If yes to Q 404, can you mention some of the problems?	1. Severe vaginal bleeding<br>2. Hypertension<br>3. Prolonged labor (>12 hours)<br>4. Placenta not delivered 30 minutes after baby (Retained placenta)<br>5. Other (specify)---------------------		
406a	In your opinion, could a woman die due to the mentioned problems?	1. Yes<br>2. No<br>3. Don't Know		
407a	Do you know any problems that can occur during the 1st week after delivery?	1. Yes<br>2. No		
408a	If yes to Q 407, can you mention some of these problems?	1. Severe vaginal bleeding<br>2. Hypertension<br>3. Fit<br>4. Swollen hands/face<br>5. High fever<br>6. Offensive vaginal discharge<br>7. Other (specify)---------------------		
409a	In your opinion, could a woman die due to the mentioned problems?	1. Yes<br>2. No<br>3. Don't Know		
410a	Do you think every pregnant woman need a skilled attendant at delivery?	1. Yes<br>2. No<br>3. Don't Know		
411a	If yes to Q 410, what is the advantages of having a skilled attendant at delivery	1. Prevention of delivery complications<br>2. Better care for new born<br>3. To get health information<br>4. Other (specify) ---------------------		

412a	If No to Q 410, why?	______________________________<br>________________________		
**B. Attitude statement**				
413b	Some people believe that any pregnant woman can develop delivery complication	1. Agree<br>2. Disagree<br>3. Indifferent		
414b	Some people feel that delivery complications can be dangerous to the health of a woman	1. Agree<br>2. Disagree<br>3. Indifferent		
415b	It is believed that delivery complications can't be dangerous to the health of the new born	1. Agree<br>2. Disagree<br>3. Indifferent		
416b	According to some people's belief a woman should plan ahead of time where she will give birth to her baby.	1. Agree<br>2. Disagree<br>3. Indifferent		
417b	Some women feel that they shouldn't plan ahead of time how they will get to the place where they will give birth.	1. Agree<br>2. Disagree<br>3. Indifferent		
418b	Some women feel that every pregnant woman need a skilled care at delivery	1. Agree<br>2. Disagree<br>3. Indifferent		
419b	Few women feel that being attended by male health personnel during delivery is unethical and shame	1. Agree<br>2. Disagree<br>3. Indifferent		
420b	According to the feeling of some pregnant women it is very shameful to deliver on delivery bed in labor ward	1. Agree<br>2. Disagree<br>3. Indifferent		
421b	Many women believe that women do not go to a health facility for delivery, mainly because it is too expensive.	1. Agree<br>2. Disagree<br>3. Indifferent		
422b	Many women believe that women do not go to a health facility for delivery because health personnel do not treat them respectfully.	1. Agree<br>2. Disagree<br>3. Indifferent		

PART V: - Factors Related to Health Institution

Ser. No.	Questions	Response	Skip	Coding
**For those who deliver in the health facility**				
501	Is there health facility in your area?	1. Yes<br>2. No<br>3. Don't know		
502	If yes to Q 501, how far is it from your house?	_________ minute		
503	If yes to Q 501, are there skilled health professionals who provide delivery service?	1. Yes<br>2. No<br>3. Don't know		
504	If yes to Q 503, have you received delivery service from this institution?	1. Yes<br>2. No		
505	Did you pay for the service you received from health institution?	1. Yes<br>2. No		
506	If yes to Q 505, how do you see the amount you paid for the service?	1. Too expensive<br>2. Fair<br>3. Cheap<br>4. I didn't remember		
508	Did you pay for the transportation?	1. Yes<br>2. No		
509	If yes to Q 508, how did you see the amount you paid for the transportation?	1. Too expensive<br>2. Fair<br>3. Cheap<br>4. I didn't remember		
510	Did you experience long waiting time to receive the delivery service?	1. Yes<br>2. No		
511	Do you have confidence on the delivery service provided at the health unit?	1. Yes<br>2. No		
512	Were the health workers respectful?	1. Yes<br>2. No<br>3. I don't know		
513	Were there measures taken to assure your privacy during the procedures?	1. Yes<br>2. No<br>3. I don't know		
514	Were you satisfied by the care given to you in the health institution?	1. Very Good<br>2. Good<br>3. Fair<br>4. Poor<br>5. Very Poor		

PART –VI: Questions on Income of the Respondent and Environmental Health Issue

Ser. No.	Questions	Response	Skip	Coding
601	What is your average monthly income (if applicable)	___________ Birr		
602	Annual harvest of each crop in quintal	1. Wheat ________<br>2. Barely ________<br>3. Teff __________<br>4. Maize ________<br>5. Bean _________<br>6. Sorghum_______<br>7. Pepper ________<br>8. Other specify__________		
603	Do you have domestic animals?	1. Yes<br>2. No		
604	If yes to Q 603, how many?	1. Cow ________<br>2. Ox _________<br>3. Sheep_______<br>4. Goat________<br>5. Horse _______<br>1. Donkey______<br>2. Other (specify) ________		
605	What is the main source of drinking water for members of your household?	a. Piped into dwelling . . . . 1<br>b. piped to yard/plot . . . . 2<br>c. public tap/standpipe . . . . 3<br>d. borehole . . . . 4<br>e. Protected well . . . . 5<br>f. Unprotected well . . . . 6<br>g. Protected spring. . . . . …..7<br>h. Unprotected spring . . . . …8<br>i. Rainwater . . . . .9<br>j. others………………………….96		
606	Do you share this toilet facility with other households?	1. Yes<br>2. No		
607	How many households use this toilet facility?	____________________		
608	What kind of toilet facility do members of your household usually use? If the respondent does not understand which type of toilet they have, ask to observe the toilet facility and circle the appropriate code.	1. Flush …………………..1<br>2. Ventilated improved Pit (VIP).. 2<br>3. Pit latrine with slab . .. . 3<br>4. Pit latrine without slab/Open pit ... 4<br>5. No facility/bush/field . . . . .5<br>6. Other (specify)……………….………6		

Name of the interviewer-________________________

Date ____________________________

Signature ________________________

Thank you!
